# Speeding up to keep up: exploring the use of AI in the research process

**DOI:** 10.1007/s00146-021-01259-0

**Published:** 2021-10-15

**Authors:** Jennifer Chubb, Peter Cowling, Darren Reed

**Affiliations:** 1 Department of Computer Science, Digital Creativity Labs, University of York, York, United Kingdom; 2 Digital Creativity Labs, Queen Mary University of London, London, United Kingdom; 3 Department of Sociology, Digital Creativity Labs, University of York, York, United Kingdom

**Keywords:** Futures, Artificial intelligence, Impact, Science, Research policy, Productivity, Academia

## Abstract

There is a long history of the science of intelligent machines and its potential to provide scientific insights have been debated since the dawn of AI. In particular, there is renewed interest in the role of AI in research and research policy as an enabler of new methods, processes, management and evaluation which is still relatively under-explored. This empirical paper explores interviews with leading scholars on the potential impact of AI on research practice and culture through deductive, thematic analysis to show the issues affecting academics and universities today. Our interviewees identify positive and negative consequences for research and researchers with respect to *collective* and *individual use*. AI is perceived as helpful with respect to information gathering and other narrow tasks, and in support of impact and interdisciplinarity. However, using AI as a way of ‘speeding up—to keep up’ with bureaucratic and metricised processes, may proliferate negative aspects of academic culture in that the expansion of AI in research should assist and not replace human creativity. Research into the future role of AI in the research process needs to go further to address these challenges, and ask fundamental questions about how AI might assist in providing new tools able to question the values and principles driving institutions and research processes. We argue that to do this an explicit movement of meta-research on the role of AI in research should consider the effects for research and researcher creativity. Anticipatory approaches and engagement of diverse and critical voices at policy level and across disciplines should also be considered.

## Introduction

The rise and convergence of technologies such as Artificial Intelligence (AI) is shaping the way we live our lives in profound ways (Brynjolfsson et al. 2019; Mazali 2018; Park 2018). Concerns over the efficacy of Machine Learning (ML) and AI approaches in a range of settings affecting the social world such as in healthcare and education (Baum 2017; Tsamados et al. 2021) make the ethicisation and governance of AI (Bryson 2019; Mittelstadt et al. 2016) a matter of pressing concern.

These concerns extend to university research. Major funders of academic research have begun to explore how AI could transform our world and the role they can play in utilising AI as an enabler of new methods, processes, management and evaluation in research (Cyranoki 2019; UKRI 2021). At the same time there is recognition of the potential for disruption to researchers and institutions (Procter et al. 2020; Royal Society 2019) and clear challenges ahead. The growing emphasis on AI creates space for empirical research to shed light on the possibilities and challenges for researchers who play a key role in developing and applying AI for wider society.

Within current debates about the future of AI and human society, AI is considered in education (Aiken and Epstein 2000; Serholt et al. 2017) and digital learning (Cope et al. 2020) but less is understood about the effects on research more broadly. Indeed, few have explored AI as an enabler of new research methods and processes, and forms of management and evaluation and there is little empirical investigation of the academic response.

The role of AI within research policy and practice is an interesting lens through which to investigate AI and society. Drawing on interviews with leading scholars, this paper reflects on the role of AI in the research process and its positive and negative implications. To do this, we reflect on the responses to the following questions; “what is the potential role of AI in the research process?” and “to what extent (to whom and in what ways) are the implications of AI in the research workplace positive or negative?”.

## Contemporary research and AI

Research is global, fast paced, competitive and there is increased expectation to do more. The UK government targets investment of 2.4% of GDP in R&D by 2027 and 3% in the longer term. A new UK government roadmap^1^ sets ambitious targets for UK science and there has been rapid growth in AI research. NESTA (2019) reports on the recent evolution of research that 77% of the papers they identified for their work on AI research were published in the last 5 years.^2^ AI is implicated in researcher efficiency and productivity (Beer 2019). A performance focussed culture can use AI in pursuit of bureaucratic aims (Ball 2012) even though this might prove detrimental to individual identities and collective scholarly norms. While the deployment of AI might work toward satisfying funder expectations of research, increasing productivity, impact and interdisciplinarity (at least according to superficial metrics), it might also sacrifice the traditional roles of institutions and academic identities. With the advent of what was termed ‘Industry 4.0’ (Kagermann et al. 2013)—a revolution in which AI will be central (Schwab 2017)—now is the time for HE to seriously consider the responsible innovation and ethics of AI in research practice, culture and HEI governance (Samuel and Derrick 2020; Samuel et al. 2021).

Research is vital for the economy and its social characteristics also extend to creating benefits for wider society and culture (UKRI 2020; Bacevic 2017). Academic research shapes academic culture (Wellcome 2020), informs teaching (Ashwin et al. 2020), identifies new areas of knowledge (Gibbons 2000) , and fills gaps in existing knowledge (Holbrook and Hrotic 2013). The role AI could play in research adds a level of complexity to a system and the academics entrenched in its habits (Bourdieu 1988). AI potentially relieves researchers and institutions from mundane tasks, which saves time (AJE 2018) and arguably boosts speed and efficiency required in a (contested) market-driven university. Yet AI also presents concerns in how the use of AI in peer review introduces bias (Checco et al. 2021; Lee et al. 2013), how AI could miss nuance and surprise (Beer 2019) and how infrastructures developed for AI in research (such as in research management), could be used for surveillance and algorithmic management (Beer 2017; Williamson 2015). Building on Beer’s (2018) “visions of cultural speedup” as a result of the expansion of data-analytics and AI algorithms, we extend this to consider the effects for research creativity.

## Current debates

The micro level of research has been less discussed and some similarities can be drawn from the effects of metrics (Wilsdon et al. 2015) and the need for responsible indicators e.g. The San Francisco Declaration on Responsible Metrics (DORA) and Leiden Manifesto (Hicks et al. 2015). The research funding community (e.g. Research Council of Norway) have been using Machine Learning (ML) and AI techniques within the research funding system (in grant management and research processes) to increase efficiency. However, further steps are needed to examine the effects and to understand what a responsible use of ML and AI would look like. The research policy community is aiming to develop and test different approaches to evaluation and allocation of research funding, such as randomisation and automated decision-making techniques.

A recent review by UKRI provides a very clear steer on the role that research can play in ensuring a beneficial future with AI, suggesting that there is potential for “AI to allow us to do research differently, radically accelerating the discovery process and enabling breakthroughs” (UKRI 2021, p.19). The Royal Society and cross-party think-tank Demos (2020) have conducted work with The Turing Institute into ways in which AI approaches are being used to aid scientific research processes. Funders led by The Global Research on Research Institute^3^ (RORI) convened to discuss AI as an enabler in research. Funders at the forefront of adoption of AI include the application of grant reviewers from the National Science Foundation (NSF) in China and the Russian Science Agency. The countries that have seized AI with the most enthusiasm are those with major issues in terms of scale and volume of research (Viglione 2020; Wilsdon 2021). In that context there is focus on positive outcomes and possibilities of AI. In addition, there is increased focus on the ethical pitfalls of AI across the world and in establishing design principles and guidelines (Bryson 2016; Hagendorff 2020; Jobin et al. 2019). It is easy for the focus on positive outcomes to be coloured in the West where there is an assumed preference for human based decision-making approaches through peer review—perhaps the least imperfect of a range of approaches (Wilsdon 2021). Bearing cultural factors in mind, little is actually asked about what changes would be beneficial. Care is needed to avoid approaching this question with the assumption that all is working well without pausing to criticise assessment, metrics, the application of narrow criteria in indicators for impact, research integrity, reproducibility for narrowing diversities, for encouraging systemic bias against types of research or researchers, or diverting attention toward only that which is valued or trackable rather than what is precious and valuable (Chubb 2017).

The debate is about what we mean by efficiency and productivity in research and whether *speeding up—to keep up*, is epistemically good. Reminiscent of an ‘accelerated academia’ (Martell 2017; Vostal 2016). While AI is seen as having huge potential to support interdisciplinary knowledge exchange, there may be deeper effects of using AI to further research policy and funders’ agendas. These may challenge traditional notions of a university and what it means to be an academic (Chubb and Watermeyer 2017; Clegg 2008; Harris 2005) which may or may not have ‘good’ consequences.

This empirical paper first provides context for the role of AI in the research landscape of the UK. A literature review of the existing research on AI in science and research is followed by an account of the methods. This paper reflects on the deductive thematic analysis of interviews with leading scholars who were asked about the role they could see AI playing in the research process. The paper aims to provide an empirical account of academic views on the potential deployment of AI in research. Authored by an interdisciplinary team of researchers (philosophy, computer science and social science) this paper aims to contribute to understanding about the broader societal and cultural impacts on higher education and research from which we hope to promote and engage academics and policy in a broader debate. The findings with respect to individual and collective benefits and concerns for research and researchers are presented and represent analysis of interviews of AI futures scholars from a range of fields. The implications for the thoughtful implementation of AI in the research process are discussed and suggestions for further research are then made.

## Defining AI

AI is often described as an ‘umbrella’ term for a range of approaches to solve data-in-data-out problems which are usually presumed to require intelligence when solved by humans and other animals, distinct from deep and machine-learning techniques which are subsets of AI. AI operates on data to generate new data which solves a pre-specified problem. Hence, AI captures a very wide range of technologies applied to decision-making problems in natural language processing, forecasting, analysis and optimisation with a range of interpretations of data as things such as speech, video, robot movements, weather forecasting and human purchasing behaviour. AI does not include deeper human and animal abilities such as the creation of meaning, the connection with others and the ability to feel and think, except where aspects of meaning, connection, feeling and thinking can be encoded as data-in-data-out decision problems. Research such as Bostrom (2017) refer to AI developments to date as ‘narrow AI’, postulating a human level of decision making: Artificial General Intelligence (AGI) considering the (probably distant) possibility of Artificial Superintelligence. Many of our participants felt that speculation over whether the latter was possible was distracting from the pressing current issues of AI usage.

## Exploring the use of AI as a research tool

The literature presents the use of AI in research as posing both opportunities and challenges. There is excitement about the opportunities AI brings for analysing large amounts of unstructured data (Van Belkon 2020) , increasing the amount of data-based research which can be performed, providing community impetus to the curation of big scientific datasets, and simplifying and speeding up the research process. At the same time, there is concern about the stability of academic skills and jobs, coupled with a sense that traditional norms of academic knowledge production might be at risk (Bryson 2016). Much literature relates to how AI will benefit or impede forms of productivity, collectively and individually. However, the meaning of “productivity” itself is debated and is not solely limited to notions of an audit culture in HE (Holmwood 2010). With respect to research, there is no doubt that there is an increasing expectation for researchers globally to publish quickly (Powell 2016). Debates about research productivity have shifted more toward considerations of quality rather than quantity and the scholarly communication process is said to be under strain (Checco et al. 2021). True, research productivity is seen to decrease in terms of the quantity of publications and academic output (Bloom et al. 2020) yet the literature also notes an increase in quality (Hill 2018). Additionally, there is a strong public perception that the ubiquitous growth of AI will impact the future of work (Royal Society 2018a, b) and expert surveys have revealed widespread thinking that AI could outsmart humans by 2045, at least in a narrow sense (Bostrom 2017). This remains highly contested by commentators (Etzioni 2016). The rate that AI is accelerating and its potential to ‘replace’ human intelligence causes public fear (Arntz et al. 2016; Muller-Heyndyk 2018) about loss of jobs. More positively, AI could replace mundane tasks or those which are seen as narrow or repetitive. Hence, while some have associated this fear with the loss of lower skilled labour, others warn that white collar workers (such as academic roles) might also face competition from technology. Some argue that it is a mistake to fear the future of work and it is simply that jobs will change (Beyond Limits 2020; Reese 2019). In this scenario AI would replace only human ‘drudgery’. There is acceptance of AI if its role is limited to assisting in augmenting performance and task substitution (Grønsund and Aanestad 2020). For others, who share the opinion that AGI is imminent (and is as powerful as human intelligence^4^), human capabilities, and thereby their work roles, will be rendered obsolete. While outside the scope of this paper, this could usher in a new era of creativity and good living for the human race, if we can collectively work out how to share the bounty from AI productivity. In the context of research, the threat of AI to jobs is feasible though there are distinct issues when we consider the nature of academic work, its history, and its values.

Research into how AI and digital technologies will impact research and science culture is relatively early stages (Checco et al. 2021). In addition to debates concerning the role of AI in productivity and the future of work, the use of tools to assist with other aspects of academic life is gaining traction. Publishers have piloted AI tools to select reviewers, check the efficacy of papers, summarise findings and flag plagiarism (Heaven 2018). Other tools like ‘AIRA’—an open access publisher’s AI assistant—generate recommendations to help assess the quality of manuscripts (Dhar 2020), and while AI to support journal editors has reduced the duration of peer review by 30% (Mrowinski et al. 2017) the outcome remains with the editor. Skepticism over the use of biased AI tools to conduct reviews is regularly described in the literature (Checco et al. 2021) whereas AI to identify discrepancies or errors is better received i.e. for use with respect to compliance or plagiarism. For instance, an AI tool 'statcheck' developed by Nuijten et al. (2016) revealed that roughly 50% of psychology papers included statistical errors. Such benefits continue to be debated alongside concerns that AI in peer review will simply reinforce existing biases (Checco et al. 2021; Derrick 2018) and the impact of using machine-learning in peer review or to guide research funding continues to be debated (Spezi et al. 2018; Weis and Jacobson 2021). There is some way to go before such tools replace a human evaluator. Studies consistently describe AI as a ‘risky fix’ and view it as a ‘runaway process’ in science (Hukkinen 2017). The Alan Turing Institute and The Royal Society (2019), raised a number of benefits and challenges arising from the increased use of AI in science. Notably, that AI combined with access to large amounts of data could be a ‘key enabler’ for a range of scientific fields ‘pushing the boundaries of science’, helping researchers to see patterns in data, find trends, clean and classify data, bridge gaps between datasets and make highly accurate predictions from complex data (2019, pp. 2–3). The near term benefits of AI seem wide ranging, but in the longer term, AI could prompt ‘unforeseen’ outcomes, potentially leading to a reframing of disciplines, modes and methods of knowledge production (Gibbons 2000; Nowotny et al. 2003). Our paper aims to contribute to the discussion about what developments in AI mean for a future scientific research culture which might rely more on digital technologies to enhance the research process and environment.

## Methods

The paper reports on the findings from (*n* = 25) interviews with leading academics working in AI futures or the applications of AI to human creativity from a range of disciplines (Table 1) from the UK, Europe, Canada and the US. Scholars were contacted following a review of the literature for recently published works in the area of AI and futures. Their responses to a question on the role of AI in research from the perspective of scholars was then deductively and thematically analysed.

**Table 1:** Participants grouped by cognate field and by discipline (*n* = 25)

Physical, natural and life sciences (8)	Computer science, electrical engineering, AI, graphics and robotics, informatics, human–robot interaction
Social science and related disciplines (12)	Geography, education, educational psychology, futures research/STS, business, psychology, race and gender studies, sociology
Arts and humanities (5)	English literature, philosophy of science, music, digital and interactive storytelling, theatre and film

Interviews were conducted face-to-face online and analysed during the Covid-19 pandemic. Interviewees were identified following a comprehensive review of the literature on AI futures and the impact of AI, and a mapping of the AI research landscape institutes, centres and universities. Following the (non-exhaustive) mapping exercise of websites, university pages, and social media, and consultation across the research team and wider academic community within the institution, it was decided that it would be prudent to consider ‘futures research’ within the context of the domain of use of AI (Börjeson et al. 2006; Jenkins et al. 2010). A draft interview schedule was developed and piloted locally based on three categories: home, leisure and culture. Interviewees were asked to describe the role they personally could see AI having in their workplace (in this instance, the university environment). They were then promoted to consider its use in teaching, research and administration. Responses with respect to research were deductively coded and then thematically analysed. We combined thematic analysis (Braun and Clarke 2006) with qualitative thematic data analysis (Miles et al. 2014). This paper reports on the deductive findings from one aspect of our research: the role of AI in the university workplace, with a focus on research. The effects of AI on teaching, though described regularly by participants, is not considered in this paper (instead e.g. see Serholt et al. 2017).

## Limitations

Interviews were conducted during a National Lockdown Summer, 2020 and this may have affected participants’ responses, at a time of multiple crisis (Simmel 2010). It can be difficult to develop a rapport with participants online and so, ahead of each interview we explained the session and offered an informal (non-recorded) chat before the main interview. We also offered interview timing flexibility, and made clear the option to withdraw or reschedule. The efficacy of such methodological practices during lockdown have been shared with the community (Jowett 2020). While generalization of an initial small scale qualitative study is difficult given the representation of disciplines, this research adds richness to existing issues and shows how AI can intersect with research from those at the cutting edge of its development and critique. The analysis was conducted across three disciplines.

Criteria for inclusion included proven expertise within AI through academic publication and current position within a research organisation/HEI. We aimed for a gender balance where possible, despite the preponderance of one gender in some disciplines. Stathoulopoulos and Matteos-Garcia (2019), report “while the share of female co-authors in AI papers is increasing, it has stagnated in disciplines related to computer science”. Despite this, 16/25 (64%) of our sample are female. Interviewees had expertise in the future of AI across a wide range of disciplines. We created a coding framework, identifying deductive and inductive codes. For the purposes of attributing participant involvement to verbatim quotation, we provide disciplinary field information and a unique numeric indicator. All interview data were anonymised at the time of analysis with individual identifiers used to denote verbatim quotations. Data were stored securely on a password protected computer with recordings deleted after use. Consent was gained for the audio-recording and transcription of interviews.

## Findings

Interviewees all commented on the prevalence of AI in research and in most aspects of modern life. AI is seen by our interviewees to have huge potential in connecting knowledge and knowledge producers, while also presenting concerns with respect to the future of work and to equality and fairness across disciplines and career stages. Below, we analyse interviewee responses related to benefits for first individual and then collective use. Then, we look at the more challenging aspects identified by participants and consider how the role of AI might disrupt academic activities. Our interviewees provide compelling arguments that whilst AI has great potential in research, it is incumbent upon actors across the research ecosystem (academics, institutions, funders and governments) to ensure that it is not used as another bureaucratic tool which further metricises and destabilises research quality and academic identities and expertise.

### AI for individual researcher use

The most commonly reported use for AI was to help with narrow, individual problems: to help researchers reveal patterns, increase speed and scale of data analysis and form new hypotheses. Many felt that the advent of web searching and online journal repositories had made it easier to ‘keep up’ with a fast moving research landscape. This was seen as transformative and was considered positive by most of our respondents. One participant argued that web searching enabled working across disciplines, enhancing their career:

*...people get very good at using search algorithms and being discerning in the results that they choose, getting up to speed on a topic very, very fast, and then being able to digest and provide the information that was needed. So that’s [a] position that only really exists because of web search algorithms, because of AI. I would say all my working life I have done jobs that have only existed because of AI, that AI being the web searching algorithms (Arts and Humanities 15).*

Almost all participants described how AI was already in use in the work environment, particularly that of higher education, in terms of enabling research practices and teaching. Several used the mode in which the interview was being conducted (online, using Zoom) to describe the everyday use of AI features:

*…to make Zoom calls have blurred backgrounds, or to make pictures look as if they are taken with an expensive lens—that is AI heavy lifting. AI—things that are doing automatic voicemail transcription and transcribing research notes - AI is great for that kind of thing (Physical, Natural & Life Science 06).*

Some commented on the role of AI and algorithms in in music or film research to boost creativity, or save time:

*I think storytelling has a role to make us see the world differently and I would love AI to be used in that direction (Arts and Humanities 17).*

*There’s lots of [AI] stuff out there now where you can press a button and you can get music in a certain style. Certain things that I use now that take me a long time, that I find clunky, so maybe certain pieces of software, I would like to be able to, you know, use in a more efficient manner (Arts and Humanities 13).*

The growing use of algorithms in research in hard to reach environments was also described:

*We’re using algorithms more now than we did even a few months ago because we can’t be present, you know, so lots of other things that we can’t check and verify, we are using models to check (Social Science 10).*

While the role of algorithms is increasing because of the volume of data they can deal with, this is largely seen as a problem orientated narrative, whereby AI is employed to solve problems. There is also a need to think about how those algorithms are interacting in particular social spheres and crucially who they affect (Noble 2018).

### AI and narrow tasks

With AI already used in research, some of which has been greatly accelerated because of the pandemic, over half of the participants then went on to describe how AI helps with narrow tasks and increases personal productivity.

*I think thinking small, thinking narrow, what are the individual problems that AI could be helpful with, we can think of just personal productivity in terms of the AI that is in your computer, helping you with search functions (Physical, Natural and Life Science 06).*

Here, AI is seen to reduce tedium and is welcomed if it is doing a very specific job or answering a specific question such as:

*Has somebody looked at this chemical before and have they found whether it will oxidise aluminium nitrate or something? and then you type that sentence in and you get competent search results coming back nicely summarised (Physical, Natural and Life Science 06).*

Several noted how AI could systematise the practice of literature searching *by* “trawling” through a lot of abstracts and then selecting those which might be relevant. Indeed, some reported the everyday use of such tools in their research team, to do ‘the heavy lifting for us’ (Engineering 06) e.g. see Paper Digest. Participants suggested that searching and summarising papers were the kinds of tasks that were ‘time consuming’, involving ‘endless drudgery’ *(Physical, Natural and Life Science 21; Arts and Humanities 04)*. While another felt that those same skills defined them as a researcher *“some people dedicate their life to learning those skills (Psychology 12).* Reflecting on Ewa Luger’s work on AI and accounting, one participant was clear in promoting understanding about the role of skills in particular professions; *“it is not about some people liking it, it is how the skill is built up about being an accountant. It is the same with radiologists, they look at for example an x-ray every seven seconds, an x-ray goes by or a mammogram goes past them, and they build up the visceral skills of understanding what to do, and what the point is, the next generation of accountants will not have that.”* Instead, Luger suggests that something else has to go in its place. They go on to suggest that *“it is not just about deskilling, it is actually understanding what those skills are before you rip them away” (Physical, Natural and Life Science 17)*.

One participant described how their subject area was so broad that filtering out the ‘wrong’ information would take years, affecting their ability to be ‘productive’.

*Right now I’m working on a meta-analysis and I’ve been going through tons and tons of papers, and it’s so dumb… Still after months of trying I still don’t have a good way to narrow it down by idea … I mean you could imagine having an AI do some of that work for you. Wouldn’t that be nice if I could? (Physical, Natural and Life Science 02).*

### AI and emotional labour

Participants referred positively to AI as long as tasks did not require ‘emotional elements’, suggesting that a line is perceived in terms of how far we are willing to accept AI into our lives and work:

*When it comes to looking at different articles etc, AI can do it way more quickly than humans can, but when it comes to making a sentence out of it or a verdict, then you also have like emotional elements attached...AI can augment human intelligence in different ways, but I don’t think we should make the mistake of trying to make them emotional because they will never really be emotional (Social Science 11).*

Participants talked about AI as a personal aid tasked with mundane, everyday research tasks, such as information retrieval:

*We are never going to have money to pay a research assistant to do this kind of [mundane information retrieval] work - it’s terrible - AI come here and do your magic (Physical, Natural and Life Science08).*

Whilst many felt using AI to sift through large quantities of data and literature was positive, they had ‘mixed feelings because this might diminish the enjoyment and satisfaction of the job,

*So, if something else could do it for me, I don’t know, but I’d feel like if somebody else could... if something else or somebody else could do that for me then I doubt that they would just stop there and not be able to do the second step as well and then I’m sort of not just out of job but out of a big passion, you know (Physical, Natural and Life Science 02).*

Other respondents were positive about working *with* AI to save time in relation to a range of niche tasks, such catching up with what their peers are doing. This might be more effective than traditional methods such as conference attendance, yet *“potentially less effective still than going to a team meeting or going for coffee with your colleagues” (Arts and Humanities 12)*. Though much of the literature about the use of AI in research relates to peer review, most of our participants did not mention it explicitly. One, however, explained that where human oversight was required, they would not welcome the use of AI.

*I cannot imagine good peer review done without humans... well, definitely human oversight but so much human intervention that it wouldn’t be that different from what’s happening now. I just can’t imagine it being possible for that to be done by a machine (Social Science 14).*

Interestingly, not a single participant mentioned the use of AI in research evaluation, another process which relies on peer review and human judgement. Where AI is making judgments, all participants expressed concern about bias. In particular, participants warned against the use of AI for decision making when a system is reliant upon training that is built on historical data. When applied to the recruitment of students and staff in universities it becomes a social justice issue.

*I would particularly worry about the application of AI in student admissions [...] I can see how there would be pressure to add AI to the mix and especially because it’s so time-consuming and its very people intensive, what I would really be afraid of is if a part of the process would be automated because what you always see when processes get automated the outliers disappear, the outliers get ignored or brushed over. I can understand that people hope to take out the personal bias of the interviewers, but it could also introduce a whole load of historical bias (Arts and Humanities 04).*

Instead participants preferred that AI should augment and assist human judgement:

*Instead of trying to copy human intelligence why not find ways and augment it instead of trying to substitute it (Social Science 11).*

One participant explained how AI could be used in the near future to bypass traditional means of knowledge production:

*I think there is certainly potential for significant speedup of research findings, I think there are certain fields that are genuinely amenable to just machine discovery of new theories, particularly very large-scale collection of new data and hypothesis generation from the data (Arts and Humanities 12).*

Many talked about how research is proliferating so fast that it is difficult for researchers to keep up:

*In academic life and in research [...] the rate at which we’re publishing stuff is exploding. We shouldn’t be doing that. But it is happening. So I think the key challenge for the future will be to navigate knowledge (Physical, Natural and Life Science 17).*

Productivity is bound to the navigation of vast amounts of research. AI is helpful if it suggests useful literature. Researchers can then train the algorithm to do better next time around. Participants remarked on the variance of the effectiveness of such tools to get to the heart of the data or information, suggestive again of the need for human insight.

### Increasing personal productivity

Participants explained that the main individual benefit of AI was the navigation of knowledge and streamlining the research process:

*I would say one area that it could possibly be useful is just streamlining the research process and helping to maybe*—*for me, it would be helpful taking care of the more tedious aspects of the research process, like maybe the references of a paper for instance, or just recommending additional relevant articles in a way that is more efficient that what is being done now (Social Science 04).*

AI was seen to help with accessing large amounts of data at speed and improving decision making by showing patterns at a scale difficult for humans to see:

*In research - with all kinds of data aggregation, you can imagine being able to sort of let an AI loose on historical data and seeing what new kinds of things can be found. On the other hand, new hypotheses that can be tested, questions that can be asked simply because then the computing power and ability will be available (Arts and Humanities 04).*

Participants referred to the fact that AI can free up time, enabling researchers to work on other things at the same time as conducting primary research. Here, participants strongly prefer AI that complements rather than replaces human expertise. This is particularly the case for certain disciplines when AI is used to discover new theories through large-scale data collection and hypothesis generation. The use of archival data in humanities research is one such area:

*I mean if [AI] could go through all the archives I painstakingly try to go through and tell me where things are, organise stuff for me in much more convenient ways to analyse historical stuff - going through my own data without me having to programme things painfully? Oh gosh I would embrace it (Arts and Humanities 04).*

Using AI technologies for individual use is where most benefit is perceived. We find that AI is welcome if it improves productivity and saves time. Specifically, AI is repeatedly viewed as beneficial for the navigation of knowledge, in the context of increased expectations to publish. When ‘let loose’ on large datasets AI has the potential to generate hypotheses and in turn increase collection and analysis capacity to streamline the research process:

*There are certain fields that are genuinely amenable to just machine discovery of new theories, particularly very large-scale collection of new data and hypothesis generation from the data (Social Science12).*

*Normally the scientific progress goes like this, so you have a hypothesis and then you collect data and try to verify or falsify the hypothesis, and now you have the data and the data, so to say, dictates you what hypothesis you can find. So, this is how methodologies, scientific methods are changing (Social Science 01).*

As scientific methods embrace AI, one participant reports the potential for things to become more complex in an already overly-bureaucratic system:

*It's a double edged sword because it has made it easier to increase the complexity of bureaucracies and forms and processes and procedures so it’s one step forward and maybe one step backwards in terms of the amount of time and energy it takes. I mean, you know, we see similar kinds of complexity (Physical, Natural and Life Science 03).*

AI might be seen to add complexity because of the steps and processes that are inherent to its design. In order for it to be beneficial, participants stressed the need to be put in the conditions to be able to benefit from it and that requires that sort of social capabilities e.g. increased understanding of the remits and capabilities of current systems and transparency. Over half of our participants spoke to the need for explainable and transparent systems that take into account the social context:

*There’s a bit of a tendency in the kind of engineering and computer sciences to sort of reduce what are quite complex things to quite simple things, like explainability, for example, or legibility. There’s a very kind of complicated social context that needs to be taken a bit more seriously, I would say (Social Science 23).*

### AI for collective use

Most participants welcomed the idea that AI could support collective activities that inform research culture and expectations, including citizen science, impact activities, and interdisciplinary working. The most cited benefits from AI were (1) its effects on modes of knowledge production, increasing freedom to conduct blue skies research; (2) facilitation of engagement and impact activities and (3) to act as a ‘bridge’ between research cultures, boosting interdisciplinarity.

### Modes of knowledge production

Participants felt that AI could release researchers to pursue new areas of ‘blue skies’ research, conduct engagement and impact activities, and work across disciplines:

*I feel the human mind and human curiosity will inevitably just be freer and more open, and quite honestly the cultural pursuits are one thing, but I really feel scientific pursuits will be another exploration, so suddenly if our world is running more efficiently, if we are not*—*you can imagine AI in policy making, eventually optimising how we use energy in the country. So, we will be able to focus more on human beauty and human knowledge. It feels like there will be more scientists who are doing basic research in trying to understand the world ourselves fundamentally (Arts and Humanities16).*

Where AI is critical in the development of new modes of research and knowledge production the key benefits are noted with respect to career stage and discipline. One participant noted the potential for ECRs to benefit from AI because of access to big datasets.

*I mean if you look at, you know, the sort of average postdoc of three years, how much data you can collect and analyse in that period could increase drastically (Physical, Natural and Life Science 11).*

Similarly, with respect to academic discipline, another participant suggested that AI could collectively benefit the arts and humanities, from which one could see a new mode of critical engagement emerge:

*I’m hoping that the idea that a text might be written by an AI rather than a human, over the next few years leads to a realisation and a new kind of critical engagement with texts where it just becomes much more the norm that people question the authorship of the texts that they read critically and that kind of critical reading is something that certainly is and should continue to be key in humanities education and I think many humanists would see AI as encroaching computers into the field, but I think there are lots of opportunities within digital humanities to use AI (Arts and Humanities 04).*

Quite how far these new modes of production might encroach on traditional disciplinary norms is not yet known but there were moments in the data suggestive of disciplinary challenges, whereby scholars might be disadvantaged by virtue of their training to benefit from AI:

*Being in a humanities department, one of the big challenges I see is helping people bridge between quite different areas of expertise. I think that’s a challenge that people are already trying to work on bringing the technology under the fingertips of students, or researchers who, by virtue of their background or interests, can’t use it themselves, but would be interested in using it. So, I see that as a major challenge (Arts and Humanities 13).*

### AI and impact

Several participants noted that AI could benefit multidisciplinary research teams with regards to open innovation, public engagement, citizen science and impact. When considering the role of AI in research, participants regularly referred to the idea that AI could act as a bridge beyond the university context and that boundaries could be expanded through greater participation in science. If used to support researchers to develop links with others and to build impact, AI could highlight the University’s civic role. As one participant described it “*communicating the potential benefits of our research to the wider world. AI can help us do that” (Arts and Humanities).* One participant thought of AI as a kind of potential co-creation tool:

*… There is a co-creation between a human author and AI that then creates a new type of story and what would that be and, more importantly, what are the conditions for this to be a real co-creation and not being one controlling the other or vice versa (Arts and Humanities 18).*

The release from narrow individual research tasks, mentioned earlier, was also seen to result in the time to deliver impact activities more effectively. One explained how academic research takes too long to move beyond the academy “*we are used to doing research that always takes so much longer, we don’t work on the same timescale [as potential users of the research] and it’s super frustrating.” (Physical, Natural and Life Science08).* Research impact takes time and effort and so AI’s ability to build connections could speed up the process. One participant suggested that AI could allow “*the vision of the open source community applied to AI” (Arts and Humanities12).* However, these infrastructures, once established could also be used for many more negative and intrusive purposes. Some participants warned about potential threats to expertise where over-use of AI could render academics ‘*generalists*’, which could be both positive and negative. On the one hand, it could be unhelpful at a time when the role of expertise is challenged in the public and political sphere (on the other the contrasting view that enabling scientists as generalists might also be key to making societally useful advances in science and the emergence of real evidence about public attitudes towards expertise as positive e.g. see Dommett and Pearce 2019). Commenting that many researchers might become generalists, one participant expressed concern about working across disciplines they didn’t train in. Despite this, AI was mostly viewed positively for increasing potential to work across disciplines and with publics *“the problems of discipline exclusivity and people not being able to talk across disciplines or understand each other and those barriers have really been broken down by AI” (Arts and Humanities 12).*

### Inequality, fairness and bias

Whilst impact is perceived as a benefit, some participants expressed concern over using AI to deliver impact activities with respect to inequalities and biases. One provided an example of where AI systems were used to promote awareness of HIV amongst high-risk populations. The AI selects peer leaders within communities of homeless youth to support awareness building. Concerns were expressed about “the potential intensification of existing inequalities that can happen (Social Science10). Several expressed concern over rising inequalities and felt that AI is only exacerbating unevenness in society; “the hugely and dramatically accelerating inequalities that are coming out of this” (Physical, Natural and Life Science 21), specifically that:

*AI might amplify existing social inequalities among the youth of different genders, races, socio-economic statuses (Social Science 24).*

One participant described AI as “a mirror to ourselves” (Futures 11) complete with biases. Generally, AI bias occurs because the data we put in are biased, due to human decisions in data curation and collection. Several interviewees suggested research to focus on explainability, trust and fairness. An important consideration therefore is *who* benefits from AI research and usage.

*Even more important is the common understanding of how we can create AI systems that are ethically responsible* (Social Science 11).

### AI and interdisciplinarity

Our research suggests that AI has potential for boosting and supporting interdisciplinarity. Overwhelmingly, participants saw real potential for AI in bridging disciplines, which could also reorientate research priorities. For instance, AI can ‘match-make’ people across disciplines.

*Some abolition of disciplinary boundaries, some significant massive participation of subjects of study in the design and carrying out of research that is affecting their lives and hopefully pretty soon a reorientation of research priorities to better match what people are generally interested in (Arts and Humanities12).*

References to AI as match-maker were common in our interviews: *“in a world where you’ve abundant and diverse interests and abundant and diverse high quality information sources, the trick is matchmaking” (Arts and Humanities 12).* One participant notes that using AI to match-make academics, would require consideration of buy in, trust and privacy: *“making such connections could happen but it requires engagement from different actors in the sector “a world that is more kind of embedded in a multistakeholder conversation, of course that’s the utopian vision, there is also a dystopian [side] to it” (Social Science 19).* The opportunity is large, as AI could greatly extend the boundaries of collaboration (Lee and Bozeman 2005). Participants noted:

*My positive vision is that we adopt some version of extreme citizen science where the boundary of who gets to contribute to research is significantly opened up, where everything is much more modular: there is a cloud of hypotheses, a cloud of data, a cloud of finding ways of connecting these to much better language, very good training, lots of ways of recruiting volunteers or collaborators to whatever interesting project that you have (Arts and Humanities 12).*

*I suppose one of the benefits I’m already seeing which I think is really advancing quite quickly is the kind of interdisciplinary collaboration, working much more closely now with computer scientists and mathematicians and physicists in my university than I did before (Social Science 10).*

This gives rise to the challenge of tailoring AI training to the needs of researchers with a wide range of disciplinary backgrounds.

### The academic role

Another theme related to how AI could affect the academic role. One participant talked about how the shifting landscape of HE had the potential to challenge traditional roles:

*If we are moving into a world where everyone is a continual learner and potentially everyone is a teacher (maybe not everyone but certainly many more people than we can think of as traditional professors), then the challenge becomes matchmaking. And having that matching be done by AI systems is probably the way you would need to go partly because this is not something that humans have traditionally been very good at and also because the scale is huge. (Arts and Humanities 12).*

They go on to envision a rather exciting future, a “*trusted ecosystem*… *matchmaking learners and academics where the academic part will probably be heavily augmented with AI”.*

Arguably, every skilled profession is in a state of evolution requiring continual learning, changing academic modes of interaction, roles and career trajectories. Some participants welcomed this enthusiastically and felt that AI will not take away from researchers and educators, but instead create new roles:

*In AI-supported learning environments, there’ll be even more need for educators and teachers and teaching, even more need than ever before. In many more places, not just schools and universities, but workplaces, community organisations, so people who are…people who facilitate any community of practice in an online environment are involved in knowledge management, learning organisations, in a broad sense (Social Science 09).*

The same participant went on to suggest that AI would lead to new forms of labour *“a profoundly modern job… and a new economy” (Social Science 04)* where AI could transform worklife for academics in positive ways. Another participant noted that whilst AI is often associated with low-skilled labour, many of those tasks which AI can and will perform are currently seen as skilled labour and higher-prestige white collar jobs are susceptible to automation: *“it is interesting that some of the higher-prestige white collar jobs are maybe more susceptible to automation than something like a food delivery service” (Physical, Natural and Life Science 06)*. Such comments are exemplified by suggestions for automating aspects of research. One participant suggested: *“we should organise the university like a gym with an app” (Arts and Humanities 17).* Participants referred regularly to the use of AI to help university functionality more generally such as building maintenance, cleaning, food selection, finances and logistics. All of these use cases have the potential to indirectly and positively affect research.

### The future of academic labour

The potential of AI to alleviate work pressure comes with an associated paradox, in which personal gain requires a sacrifice of privacy through the gathering of large amounts of data on individuals:

*You could imagine a university of the future where there would be much, much, much more data on people and much more understanding of how they learn… I have mixed feelings about it (Physical, Natural and Life Science 02).*

When imagining a use for AI in the context of any domain of (human) work, there were concerns about the loss of jobs. In particular, this was seen to threaten certain groups, including early career researchers and researchers from the arts and humanities:

*We’ve seen the hiring of fewer and fewer staff in terms of research within the humanities (Social Science 25).*

Participants commenting on the use of AI in the humanities suggested that human knowledge will still be required alongside AI:

*There will still be people who are studying urban planning, even though there are urban planning AI*—*there will be people doing that. If [the AI agents] are doing it better than us, fine*—*we will have scholars preserving the human knowledge and then pointing to why the AI knowledge is so much better. It just comes down to ego or not, in that case. (Arts and Humanities 16).*

The implicit challenge of the word ‘better’ in this case provokes debate about the role of metrics in HE. The need to ensure that ‘unemotional’ AI only compliments and does not replace human knowledge with ‘deep information’ is particularly pertinent to certain collective groups, such as the arts and humanities who may find it difficult to objectively measure their cultural value and impact (Benneworth 2015; Belfiore 2015):

*With budget cutting scenarios, I wonder to what extent various kinds of programmes that don’t fully work will be used to justify attrition of things that are currently done by people (Social Science 25).*

Despite relative confidence among our participants that AI will not replace established academics, AI is seen to potentially challenge more precarious groups. Whilst AI is presented here both in positive and negative terms, it is already in use and we must now deal with the ‘hangover,’ as one participant aptly put it:

*I joked on social media that we had our big party on Saturday night and now it’s Sunday morning and we got a hangover and we’re sobering up and we’re saying wow, there are some great potentials for bad and terrible uses of technology as well as very good ones (Physical, Natural and Life Science 03).*

Anticipating the good and bad effects of AI will surely be key to better ensuring benefit which helps humanity to thrive, rather than impedes it. This is perhaps particularly pertinent during times of crisis.

*If AI is let’s say replacing human capitalistic work in certain ways, the question I would like to ask is how is it making our lives better? And, one of the things that we will likely be holding on to*—*at least for the near future, near to medium-term future*—*is human creativity and culture. So, everything from religion to art and performance, the human spirit I feel will be the last thing for us to stop appreciating (Arts and Humanities 16).*

We now draw together the findings in a discussion of the challenges and benefits perceived by our participants and explore their effects supported by argumentation in the literature.

## Discussion

**Fig. 1:**
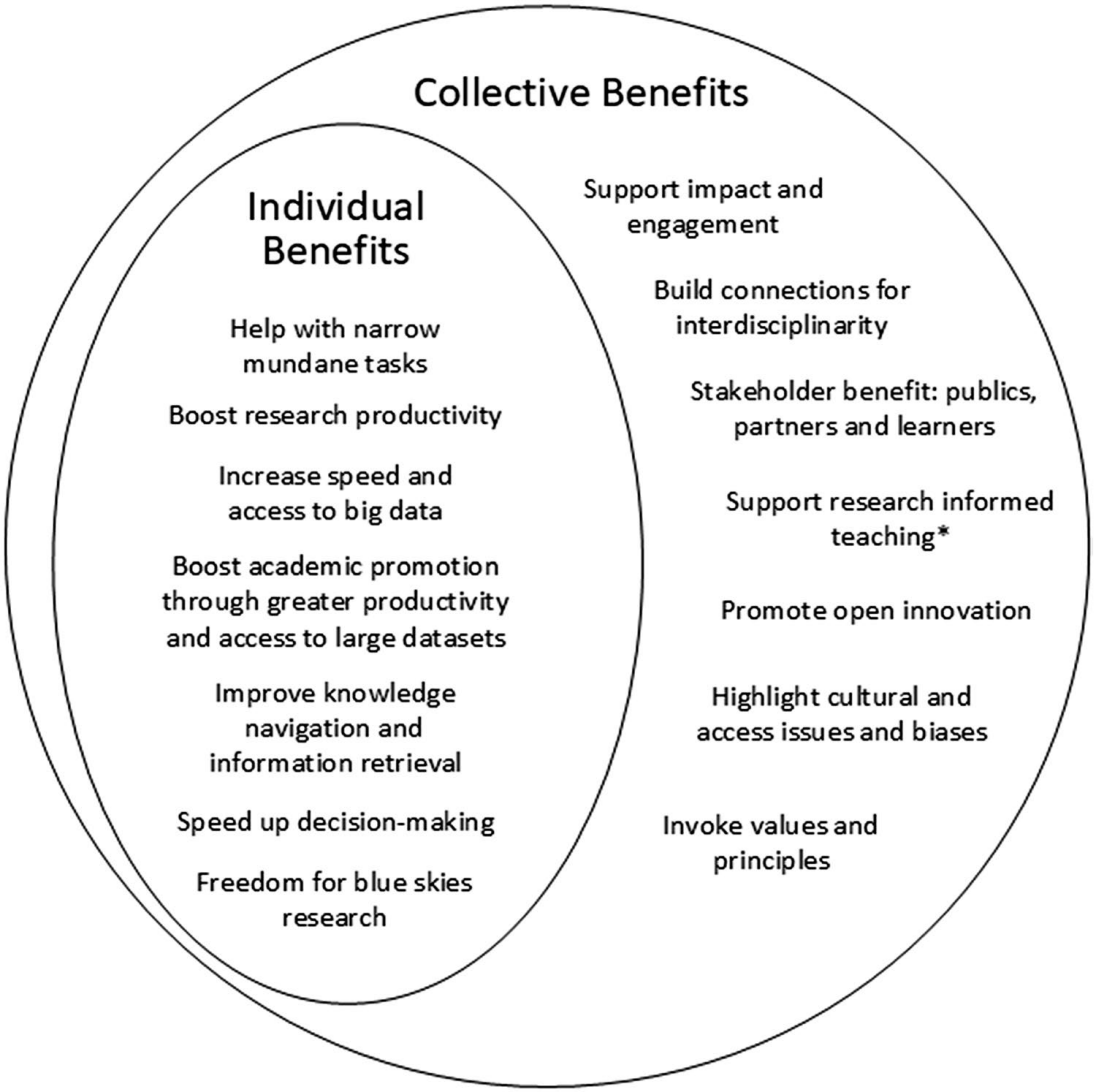
Individual and collective benefits of AI in research from thematic analysis of (*n* = 25) participants. *AI in teaching was excluded from the discussion of this paper

We draw together the findings in a discussion of the challenges and benefits perceived by our participants and explore their effects as illustrated in Fig. 1.

The views and experiences of this study’s AI thought leaders help formulate an understanding of the current position of AI in research and likely routes forward. In general, our interviewees perceive the benefits of AI to be focussed on individual tasks such as the navigation of large data sets, particularly volumes of text, as well as providing collective benefits for groups across disciplines through facilitation of collaboration and impact. Primarily interviewees constructed their responses around the benefits, in line with the interview prompts to consider the opportunities for AI. Nevertheless, the possibilities of AI present challenges which require deep reflection, reminiscent of related debates in research about academic productivity, metrics and algorithmic allocation (Arruda et al. 2016; Bonn and Pinxton 2020; Bornmann and Haunschild 2018; Dix et al. 2020; Wilsdon et al. 2015, 2017). There was optimism about the way AI might relieve tedium and open up new knowledge pathways. But this was coloured by concerns that AI tools used unthinkingly might promote bias and inequality. AI is seen to potentially challenge more precarious groups (Gruber 2014; Herschberg et al. 2018; Ivancheva et al. 2019). There is a preference for AI to play a role within research that assists rather than replaces human performance. In this section we discuss key themes which were discussed by our interviewees and relate them to the university context before using them to suggest a route forward.

### Contemporary research and AI

The contemporary research context is a product of a complex set of factors, including the marketisation of the university and the multiplication and fragmentation of research areas. Asked to think about a future with AI in the workplace—in this case the research environment—participants described what the university of the future might look like and what role AI could play in it. Against a backdrop where increased investment in research demands greater output and impact, notions of productivity are increasingly tied to performance metrics and bureaucratic processes of a ‘performative’ university culture (Ball 2012). Thoughtless application of AI could “speed up” research to “keep up” with the metrics while negatively impacting the quality of research and the quality of life of researchers. Increasingly, research income is allocated at scale to underpin the UK ‘knowledge economy’ and to firm up the UK’s position in the world as a global leader of science and research (Olssen and Peters 2005). There is broad consensus that the total investment in research in the UK will increase (Royal Society 2019^5^). The UK Government has committed to a target of 2.4% of GDP invested in research and development by 2027, with a longer term goal of 3%. Increased investment will flow into research through the science budget via research councils, the block grant resulting from the REF research audit and direct funding of large centres and projects, as well as through industry investment. Accountability and measured performance are a condition of funding, for instance, the requirement to demonstrate the impact of research through The Research Excellence Framework (REF) and grant funding enables institutions and funders to make ‘value’ visible to the government. Though rather knottily related to the question of how you measure research productivity, research grants and systems to assess the quality of UK research have been reported as damaging to traditional notions of what universities are for (Collini 2012; Martin 2011; Chubb and Watermeyer 2017). One consequence of AI undertaking narrow and mundane tasks is that it makes space and time to pursue other forms of collective knowledge production (Gibbons 2000). What Van Belkom (2020a) refers to as “naive” but “breakthrough” research. Against this background, metric-driven AI tools will need thoughtful management to avoid a situation of rising scores and declining quality, value and researcher wellbeing.

### Productivity

As with many areas of contemporary society, the university is reliant upon information technology. The incorporation of AI and machine learning are a logical next step. AI is an engine of productivity and the need for information navigation tools is a consequence of a rapid increase in research production and information accumulation. Productivity in this sense is an issue faced by contemporary academics. They are expected to be productive and at the same time cope with the increasing volumes of output of others. Interviewees referred to increased demands in academia to produce and to be seen to be productive. This begs the question as to what being “productive” means. The data points to a deeper issue about integrity and the need for academics to establish the true value of their work as opposed to what is likely to satisfy institutional and funder requirements (and of course universities and funders should continue to attempt to steer toward true value). Importantly, to approach the research system with the assumption that it is working diverts attention to only that which is valued, or trackable, rather than what is most precious to the researcher and to society. AI is perceived by our participants as reinforcing individual and selfish behaviours in pursuit of particular targets or measures (Elton 2004). There is a danger in considering productivity in terms of precisely-defined metrics (e.g. REF). AI is a very effective tool for ‘measuring’ productivity. However, our data supports the continued need for human judgement in decision making. A focus on efficiency and productivity—*speeding up to keep up* with a fast moving knowledge base—might therefore weaken output quality as it obscures the use of human judgement to produce unexpected interpretations of data and literature. In turn, this might encourage deleterious consequences for particular individual and epistemic identities (Chubb and Watermeyer 2017). One theme, explicitly and implicitly made in the interviews was that AI will never be emotional. Research roles that require emotional and nuanced judgement, such as forms of research evaluation, should avoid AI. The idea that research quality and productivity pull in different directions is a cause for concern, and a crucial issue for funders, research leaders and researchers in Science and Technology Studies, Research Policy, AI Futures and ethics to address.

### Impact, engagement and AI

AI is beneficial when it supports information navigation and knowledge production. This occurs in the daily practices of researchers in web searches and communications technologies. It supports connections between researchers. Interviewees described how there may be collective benefit to using AI in research to connect researchers from different disciplines whilst others warned about how this may lead to academics being generalists and not experts. The impact agenda requires increased public engagement and interaction (Reed et al. 2014), which might in turn encourage generalism. But public intellectualism is perhaps better understood in terms of accessible specialisms (Collini 2012). At the same time, a new era of scientific generalists may spark a renaissance in science as ideas travel across disciplinary cliques and into public attention. While clear benefit can come from championing the role of AI to boost impact, disciplinary differences need to be sensitively considered. Some disciplines and groups, such as the Humanities and ECRs, may be disadvantaged, reducing the need of student support, and undermining valuable career experience. Recognition of AI as a component of ubiquitous computing systems, such as web search and recommender systems, is useful. The explicitness of naming something ‘AI’ reinforces the ‘good’ use of AI as its ability to speed up and make research more efficient. But this is problematic where a culture of performativity is damaging to individuals and groups. Responsibly employed AI could strengthen meaningful relationships within and between disciplines as well as between academia and the public.

### Interpretations of work and emotion

The assumption that processing data is a large part of a researcher's job provides a reductionist approach to the academic role. A human reduced to any number of data points is a poor facsimile. The same being true for human organisations. Rather, humans are agents of *meaning*—the reader of a scientific article does not simply “process” the words of an article. Instead, they interpret, they experience. With this in mind, to assume that Science is about creating data, tells only a fraction of the story. Much more important is creativity and human understanding. We note how interviewees instead referred to AI as taking away or relieving them from the ‘drudgery’ of certain tasks, however, several warned that this was not reductionist and that a range of roles, research assistants, archivists etc., within research, and a range of skills gained through training as a researcher, could be lost or replaced by automation and AI. The skills accumulated when sifting through information might be critical to the person’s role as a generator of meaning. Luger’s (2021) current work ‘Exploring Ethical AI in Accounting’ provides an example. In her study of accountants Luger argues that the removal of mundane work—in the case of accounting looking through receipts, for example—reduces professional skills development. Instead there should be greater consideration of understanding skills rather than just blindly taking them away or replacing them. AI should then not necessarily take those mundane tasks away from researchers, because it would take away a fundamental skill relevant to a profession. Our interviewees prefer for AI to be limited toward the factual as opposed to the interpretative, chiming with views commonly held that AI ought to assist in the workplace, i.e. *‘IA instead of AI’* (Pasquale 2020). Interviewees expressed concern about loss of jobs, particularly for those whose roles demanded more repetitive tasks. At the same time, some noted how computation could increase demand for labour. While a counter position to this may err toward a more reductionist view of the scientific enterprise, the preferred view of science, knowledge and discovery is that it is precious and should not be reduced to a series of tasks and measured by metrics. The extent to which this relates only to AI, is debatable, but a form of anticipatory governance—motivating foresight and engagement at all levels, vis-à-vis the implementation of AI within professional roles—seems appropriate (Fuller 2009).

Whilst our participants identified a clear beneficial role for AI in navigating large bodies of knowledge, information and data, there is also potential for generating impact and nurturing interdisciplinarity. AI, alongside human creativity and insight, could yield extraordinary benefits through research. At the same time, there are threats to groups of researchers where a reliance on technology destabilises certain kinds of knowledge production and producers. The replacement of human capabilities in collective activities such as peer review, where human judgement is deemed vital, is considered undesirable by our participants.

### Operationalising values in research

The interview data echoes macro level debates about human ‘values’ in research. Interviewees foresaw issues with the ways in which AI might reflect and further existing inequalities and bias. A large amount of research and regulation has targeted the minimisation of this bias (Caliskan et al. 2017; Röösli et al. 2021; Zajko 2021), but the hidden consequences of AI adoption with respect to research have yet to be fully addressed. If AI challenges institutional and academic identities and helps shape the future role of the academy, it may be pertinent to ask how technology can support, rather than impede, the process. But as AI bias originates in human beings, there is an important consideration to address e.g. as AI emerges from communities (developers and technologists, etc.), which themselves hold certain values and perspectives and priorities, which may be distinct from the users—here, academia and scholars, then the technology itself cannot be seen as value free or neutral in this process. Rather, as much of the literature shows, AI bias ought to tackle the very stories and narratives which are propagated from the fairly homogenous group from which they often come. Narratives and story can pervade public and policy perception (Cave et al. 2018; Cave and Dihal 2019). A study by the Royal Society suggests there is urgency to take AI narratives seriously to improve public reasoning and narrative evidence (Dillon and Craig 2022). What is required, they suggest, is ‘story-listening’—an active engagement and anticipation with narratives as a form of public participation. Issues of social justice emerge as key concerns within AI. As gendered AIs populate the media (Yee 2017; Cave et al. 2020) and our homes (Alexa and Cortana, etc.), questions are rightly focussed on who is telling the stories that are informing our sense-making about AI. Indeed, the extent to which these fictional narratives inform and engage with issues of race (Cave and Dihal 2020; Cave et al. 2019) and ascribe a view of ‘whiteness’ is of ongoing concern and debate, not only through stories, but in wider attempts to decolonize AI (Noble 2018; Benjamin 2019). Shining a light on these design issues, where AI might embody such values and principles needs to be evaluated with society and culture at the heart. In the case of academia, a diverse community, attention as to how these stories and perspectives are shaping this diverse community or holding back areas of science which already struggle to be so.

## Conclusion

The 2021 UKRI report *Transforming our World with AI* (UKRI 2021) suggests that the “profound impact of AI… has not yet been realised”, and that AI “can open up new avenues of scientific study and exploration”. This paper strongly supports these views by providing insights from leading AI Futures scholars. Through this we have a better understanding of the questions to be asked and actions taken to achieve outcomes that balance research quality and researcher quality of life with the demands for impact, measurement and added bureaucracy.

The effects of AI tools in scientific research are already profound. Our interviewees discussed individual and cultural factors, seeing AI opportunities in areas where change might be appreciated, alongside a desire for stability for more entrenched habitus. Some comparisons on the micro level of research policy can be made with the debate within research and responsible metrics and how to foster responsible applications of AI. AI strategy can learn from wider discussion on metrics, aiming to avoid further worsening the impact of metrics in higher education**.** Multidisciplinary academic teams should test the reliability of systems, whatever their domain of use, and this could encourage a fairer, more just, use of AI. Each potential application of AI will give rise to positives and negatives. Identifying what these positives and negatives are at a high level (e.g. their impact on early career researchers) is urgent. It is here that multiple stakeholders across the research system must exercise their agency (by deciding which systems to buy, build and use) and implement with care conscious of the lack of neutrality in technologies they seek to deploy in an already diverse community. There is also a need for futures research, anticipatory governance, and forecasting to develop a beneficial and supportive research culture where AI is part.

While AI is a tool for solving problems modelled as data-in-data-out processes, such problems represent a small fraction of human experience. The participants express concern about removing the ‘human’ from future research. Issues of interpretation, value, and principle ring out in discussions of emotional investment, fairness, and care for colleagues. It is critical to achieve quality over quantity. These concerns reassert the human character of research where research is much more about curiosity, exploration and fascination than it is about solving data-in-data-out problems. The danger and fear is that the desire for measurable research products will eclipse human considerations. We are therefore left with a choice as to how far AI is incorporated into future research and to what end. Currently, there is no clear strategy. As tools are developed they are embraced by some, rejected by others. There is insufficient information to guide best practice or consideration as to what the limits of AI application should be. It is unsurprising that the result is excitement and fear in equal measure. We need, perhaps, to step away from the relentless push towards greater measured productivity and ask more important questions about what we want for the future. These rest on a realistic view of what AI can and cannot do and a decoupling of truly effective research practice from measured research outcomes.

AI presents a challenge for research and researchers. Whilst AI may have a positive impact, the realisation of benefits relies on the actions and decisions of human users, and the research cultures in which it is designed and deployed. To find a useful and beneficial role for AI, wider stakeholder discussions are needed on the challenges posed by introducing AI into the research process and to reflect on where its use is inappropriate or disadvantageous for research or researchers. If AI is to be deployed responsibly, incentives need to be provided and there needs to be acceptance of the potential for disruption. AI might, as one of our participants stated, ‘*rock the boat*’ and there will be a divide between those that do and do not have the mindset that AI can assist in true productivity rather than simply replacing human academic labour. As discussed, knowledge production is entrenched in long standing scholarly norms and ideals and change can manifest fear among researchers, stimulating a drain of AI early career researchers to industry (and models motivated ultimately by questions of profit). There is work to do in terms of developing a research culture in which AI supports academic tasks in a way that is meaningful and edifying for researchers, and enriches the knowledge systems within which they operate. To do so, there is a need to interrogate and invoke the values and principles driving institutions. Universities can consider ways to improve the conditions for researchers to retain them.

AI can help research and researchers, but a deeper debate is required at all levels so as to avoid unintended negative consequences. This requires key players, e.g. funders, HE institutions, publishers and researchers to participate in leading and informing this debate, even leveraging initiatives such as those used to promote the responsible use of metrics in research (e.g. DORA and others), to extend into these areas. Alongside this, Global actors and signatories of consortia in Research on Research and meta-research, as well as scholars interested in the social implications of AI, must work with AI and Machine Learning scholars at all career stages and from varying backgrounds to help deepen and adapt the Responsible Research and Innovation discourse. The understanding of how AI can transform society has itself become a new multidisciplinary research field: AI Futures. Our explicit aims lay the ground for further much-needed exploration. More research is needed to establish a clear view of the role of AI in research. There is, then, a need for new narratives about the ways in which AI can support academic labour and help make sense of its introduction (Felt 2017). This includes addressing the systemic issues of research and HE and requires deeper foresight and futures research (Van Belkom 2020b).

It will be pertinent to ask if AI can help to foster change and enable responsible practices in research and how AI can help us address long standing issues in research. Stakeholders across the research ecosystem will need to identify the values and virtues they wish to see in their institutions and turn inward to address the assumptions they make and the biases they propagate. Research is needed now to avoid a situation where AI simply allows us to “speed up” to “keep up” with an ever-increasing focus on measured research outputs. In an environment of increased research power, the human capacity for deciding what questions are worth pursuing becomes more valuable than ever.

## Data Availability

Anonymised data can be made available upon request via ethics approval.

## Footnotes

1 https://www.gov.uk/government/publications/uk-research-and-development-roadmap.

2 https://www.nesta.org.uk/report/semantic-analysis-recent-evolution-ai-research/.

3 The Research on Research Institute (RORI) consortium includes 21 partners, drawn from 13 countries or regions https://researchonresearch.org/.

4 AGI. A contested term, https://intelligence.org/2013/08/11/what-is-agi/, broadly AI with human level performance.

5 https://royalsociety.org/-/media/policy/projects/investing-in-uk-r-and-d/2019/investing-in-UK-r-and-d-may-2019.pdf.

## Code availability

N/A.
